# Blood-Based Biomarkers Are Associated with Disease Recurrence and Survival in Gastrointestinal Stroma Tumor Patients after Surgical Resection

**DOI:** 10.1371/journal.pone.0159448

**Published:** 2016-07-25

**Authors:** Michael Stotz, Bernadette Liegl-Atzwanger, Florian Posch, Edvin Mrsic, Michael Thalhammer, Tatjana Stojakovic, Angelika Bezan, Martin Pichler, Armin Gerger, Joanna Szkandera

**Affiliations:** 1 Division of Clinical Oncology, Department of Medicine, Medical University of Graz, Graz, Austria; 2 Research Unit Genetic Epidemiology and Pharmacogenetics, Division of Clinical Oncology, Medical University of Graz, Graz, Austria; 3 Institute of Pathology, Medical University of Graz, Graz, Austria; 4 Division of General Surgery, Department of Surgery, Medical University of Graz, Graz, Austria; 5 Clinical Institute of Medical and Chemical Laboratory Diagnostics, Medical University of Graz, Graz, Austria; 6 Research Unit for non-coding RNAs and genome editing in cancer, Division of Clinical Oncology, Medical University of Graz, Graz, Austria; 7 Department of Experimental Therapeutics, The University of Texas MD Anderson Cancer Center, Houston, United States of America; 8 Center for Biomarker Research in Medicine (CBmed), Medical University of Graz, Graz, Austria; University of Pittsburgh Cancer Institute, UNITED STATES

## Abstract

**Background:**

Inflammatory blood count biomarkers may improve recurrence risk stratification and inform long-term prognosis of cancer patients. Here, we quantify the prognostic impact of blood-based biomarkers on recurrence risk and long-term survival in a large cohort of gastrointestinal stroma tumor (GIST) patients after curative surgery.

**Methods:**

One-hundred-forty-nine consecutive GIST patients were followed-up for a median period of 4.8 years. Local recurrence, distant metastasis, and death occurred in 9, 21, and 31 patients, respectively. Time-to-event and competing risk analysis were applied to study the association between haemoglobin (Hb) level, white blood cell count (WBC), neutrophil/lymphocyte ratio (NLR), derived NLR (dNLR), lymphocyte/monocyte ratio (LMR), and platelet/lymphocyte ratio (PLR) with risk of local or distant recurrence (RR), recurrence free survival (RFS), and overall survival (OS).

**Results:**

A low Hb (p = 0.029), and elevations in the parameters WBC (p = 0.004), NLR (p = 0.015) and dNLR (p = 0.037) were associated with a poor OS in GIST patients in multivariate analysis. Moreover, a low Hb (p = 0.049) and an elevated WBC (p = 0.001), NLR (p = 0.007), dNLR (p = 0.043) and PLR (p = 0.024) were independently associated with decreased RFS after adjusting for Miettinen score. However, only an increase of dNLR/NLR showed a significant association to higher RR (p = 0.048). Inclusion of NLR or PLR to Miettinen risk score did not reasonably improve the clinical risk prediction of 2-year RFS.

**Conclusion:**

Low Hb, elevated WBC, elevated dNLR, and elevated PLR are independent prognostic factors for a worse clinical outcome in GIST patients after curative resection.

## Introduction

Gastrointestinal stroma tumors (GIST) are mesenchymal malignancies that represent the most frequent subtype of sarcomas and are usually characterized by mutations in the KIT and PDGFRA proto-oncogenes [1–4]. GISTs can develop at any site of the gastrointestinal tract, most common in the stomach with a frequency of about 60–70 percent, followed by the small intestine (about 30 percent) [5]. The median age at presentation is 65 years, and patients typically present with symptoms such as pain, anaemia and/or bleeding. Therefore, a major part of GISTs are detected in a localized and therefore operable stage [6, 7]. Operative resection is the standard treatment in a localized stadium and should be carried out whenever feasible. Fifteen-year recurrence-free survival (RFS) after surgery alone has been reported in up to 59.9% of cases [8]. However, estimating the risk of tumor recurrence is of great clinical impact in the management of operable GIST, because in the era of adjuvant imatinib treatment, the accurate determination of the risk for tumor recurrence is increasingly important to select the appropriate patients for adjuvant therapy [8].

The introduction of imatinib in the treatment of GIST has revolutionized GIST patients’ outcome by reducing the risk of local and distant recurrence and improving overall survival. Tyrosine kinase inhibitors (TKI) are now standard treatment in metastatic GIST, and in the adjuvant setting, patients at intermediate or high risk of recurrence, should be considered for adjuvant treatment with imatinib [9–11]. At present, recurrence risk stratification relies on tumor specific parameters such as tumor size, tumor location, mitotic index and depending on the risk stratification used also tumor rupture [9]. However, the most important prognostic factor for GIST recurrence after surgery is a high tumor mitotic rate [12–14]. Based on these prognostic factors, several nomograms have been developed to help in risk stratification for achieving an optimal risk assessment [8, 15–18]. Improved prediction of RFS or disease progression (i.e. a composite of local recurrence and onset of distant metastasis) in primary GIST after curative resection is an important goal of clinical research, because it may allow directing adjuvant therapy to the patients with the highest clinical benefit while sparing low-risk patients from the unnecessary burden of additional treatment. In the last few years, the close association of malignancy and inflammation has been intensively investigated on a basic and clinical level, and novel immune-system-interfering therapies such as immune checkpoint inhibitors are in the process of revolutionizing anticancer therapy. The variation in systemic inflammatory cell amount might be a valuable pre-treatment prognostic marker for stratifying patients at risk for tumor recurrence, as this has already been demonstrated by using the neutrophil-to-lymphocyte-ratio (NLR), platelet-to-lymphocyte-ratio (PLR), and lymphocyte-to-monocyte-ratio (LMR) in various cancer entities [19–22]. In GIST, blood-count-based inflammatory markers have been investigated as predictors of survival, but the potential of these easily-available biomarkers for improving the prediction of recurrence risk and improving stratification for adjuvant therapy has not been explored. In this study, we firstly aimed to investigate whether haemoglobin (Hb) level, white blood cell count (WBC), LMR, PLR, NLR or derived NLR (dNLR) associate with local recurrence, distant metastasis, and survival outcomes in GIST patients. Secondly, we evaluated whether the addition of these parameters to the Miettinen score, a validated prediction model for adjuvant treatment selection, might improve the prediction of tumor recurrence.

## Materials and Methods

### Subjects

This retrospective analysis included data from 149 consecutive patients who were treated at the Division of Clinical Oncology and the Division of General Surgery, Medical University of Graz, between 1997 and 2014. All patients suffered from histologically confirmed GIST, and had a basic blood count available at the date of diagnosis. All clinico-pathological data were retrieved from our in-house medical records, as well as from pathology records from the Institute of Pathology at our institution. The preoperative blood cell count was obtained within 7 days before surgery and performed for routine clinical practice within the general routine laboratory of our hospital, the Barmherzigen Brüder Hospital or the Elisabethinen Hospital in Graz. Patients with signs of systemic inflammation related to infection at time of diagnosis were excluded from the study population. The LMR was calculated from the routinely performed preoperative blood cell count as the absolute count of lymphocytes divided by the absolute count of monocytes. The PLR and NLR were calculated as the absolute count of platelets or neutrophils divided by the absolute count of lymphocytes, respectively. The derived neutrophil-lymphocyte-ratio (dNLR) was calculated as the absolute neutrophil count / (absolute leukocyte count–absolute neutrophil count) [23]. Follow-up evaluations were performed every three months within the first three years, six months for five years and annually thereafter for curative resected tumor stages. Follow-up investigations included clinical check-up, laboratory and radiological assessment. For deceased patients, dates of death were obtained from the central registry of the Austrian Bureau of Statistics. The study was approved by the Institutional Review Board (IRB) of the Medical University of Graz (No. 25–458 ex 12/13). Written informed consent was obtained from all participants at time of first medical contact as component part of the “biobank program” of the comprehensive cancer center Graz.

### Statistical Analysis

All statistical analyses were performed using Stata (Windows version 13.0, Stata Corp., Houston, TX). Continuous variables were reported by medians (25th– 75th percentile), whereas count data were summarized using absolute frequencies and percentages. Spearman’s rank-based correlation coefficient, rank-sum tests, and chi-squared tests were applied to study the association between two continuous variables, between one continuous and one categorical variable, and between two categorical variables, respectively. Median follow-up was estimated with the method of Schemper & Smith [24]. OS was estimated using the Kaplan-Meier product limit estimator, whereas the cumulative incidence of non-terminal endpoints such as local recurrence and distant metastasis was estimated using competing risk estimators accounting for mortality as the competing event [25]. RFS was defined as a composite endpoint of local recurrence, distant metastasis, or death-from-any-cause, whatever came first. Two or more survivor functions and two or more cumulative incidence functions were compared using log-rank tests and Gray’s tests, respectively [26]. Uni- and multivariable modeling of time-to-death and time-to-other-non-terminal-endpoints was performed using Cox Regression and Fine & Gray Proportional Subdistribution Hazards Regression, respectively [27]. The improvement in predictive potential upon addition of blood-based biomarkers to the Miettinen score was assessed by comparing Harrell’s concordance index (Harrell’s C) between models with and without these biomarkers [28].

## Results

### Baseline analysis

One-hundred-forty-nine patients with histologically-confirmed GIST were included in the analysis. All of these 149 patients underwent surgery with curative intent, and none of the patients had evidence of distant metastasis at the time of surgery. The median age at surgery was 65.5 years (range: 13–96, Table 1). The median tumor size was 5cm (range: 1–26). cKIT immunohistochemistry positivity was present in 148 (100%) of the 148 patients with non-missing cKIT status. Thirty patients (20.3%) received adjuvant treatment with imatinib for 1–3 years according to the current recommendations. Twenty-seven (18.1%) of the 149 patients had a second primary malignancy (SPM) at any time before or during the study. Ten (37.0%) out of the 27 total SPMs were diagnosed before GIST surgery, 11 (40.7%) SPMs were diagnosed at or within 1 month around GIST surgery, and 6 (22.2%) SPMs were diagnosed during follow-up. The median time between SPM diagnosis and GIST surgery was 1854 days (25th-75th percentile: 278–3636) in patients with a SPM diagnosis before GIST surgery, 0 days (25th-75th percentile: 0–0) in patients with an SPM diagnosis within 1 month of GIST surgery, and 1630 days (25th-75th percentile: 278–3636) in patients with an SPM diagnosis after GIST surgery. Strong correlations were observed between white blood cell counts, neutrophil counts, lymphocyte counts, monocyte counts, NLR, dNLR, and LMR were highly correlated with each other (S1 Table). However, neutrophil and lymphocyte counts did not appear to correlate with each other, and the correlation between lymphocyte and monocyte counts was weak.

**Table 1:** Baseline characteristics of the study population. Continuous variables such as age are reported as medians [25^th^-75^th^ percentile], and categorical variables such as GIST location are reported as absolute frequencies (%). Abbreviations: cm–centimeter, G/L–giga per liter.

Variable	n(% missing)	Overall (n = 149)
**Demographic variables**		
Age (years)	149 (0.0%)	65.5 [56.7–73.5]
Male gender	149 (0.0%)	85 (57.1%)
**Clinical variables**		
cKIT Immunohistochemistry positivity	148 (0.7%)	148 (100%)
Tumor size (cm)	149 (0.0%)	5 [3–7]
Mitotic rate	149 (0.0%)	49 (32.9%)
Localization	149 (0.0%)	/
—Stomach	/	99 (66.4%)
—Duodenum	/	9 (6.0%)
—Small intestine	/	24 (16.1%)
—Rectum	/	9 (6.0%)
—Other	/	8 (5.3%)
Miettinen risk score	141 (5.6%)	/
—None	/	30 (20.8%)
—Very low	/	36 (25.0%)
—Low	/	20 (13.9%)
—Moderate	/	18 (12.5%)
—High	/	37 (24.8%)
Second primary malignancy (SPM)	149 (0.0%)	27 (18.1%)
—at or before baseline	149 (0.0%)	21 (14.1%)
**Laboratory variables**		
Haemoglobin (g/dL)	148 (1.3%)	12.9 [10.9–14.1]
White blood count (G/L)	149 (0.0%)	6.6 [5.4–9.1]
Absolute neutrophil count (G/L)	149 (0.0%)	4.3 [3.1–6.1]
Absolute lymphocyte count (G/L)	149 (0.0%)	1.4 [1.1–1.8]
Absolute monocyte count (G/L)	149 (0.0%)	0.5 [0.4–0.7]
Neutrophil-lymphocyte-ratio (NLR)	149 (0.0%)	2.9 [2.0–4.9]
Derived neutrophil-lymphocyte-ratio (dNLR)	148 (1.3%)	2.0 [1.4–2.7]
Lymphocyte-monocyte-ratio (LMR)	149 (0.0%)	2.8 [1.8–3.9]
Platelet count (G/L)	149 (0.0%)	252 [211–306]
Platelet-lymphocyte ratio (PLR)	149 (0.0%)	180 [129–243]

### Prospective analysis

Patients were followed for a median of 4.8 years (Range: 3 months– 13.9 years), and only 9 patients (6.0%) were lost-to-follow-up during the study period. More than 80% of the patient population was followed for more than 1 year. The 9 patients that were lost-to-follow-up were followed for a median interval of 5.7 years. Thirty-one (20.8%) patients died during follow-up. The Kaplan-Meier 1-, 2-, 5-, and 10-year overall survival rates were 95.7% (95%CI: 90.7–98.1), 92.5% (95%CI: 86.4–95.9), 78.4%, (95%CI: 68.7–85.5) and 58.5% (95%CI: 42.6–71.4), respectively (S1 Fig). The Kaplan-Meier 1-, 2-, 5-, and 10-year recurrence-free survival rates were 92.0% (95%CI: 86.1–95.5), 84.4% (95%CI: 76.9–89.7), 66.3% (95%CI: 55.9–74.8) and 50.6% (95%CI: 38.1–61.9), respectively (S1 Fig). During follow-up, 9 patients (6.0%), 21 patients (14.1%), and 4 patients (2.7%) developed local recurrence, distant metastasis, or both, respectively. The cumulative 1-, 2–5-, and 10-year recurrence rate (RR) accounting for death as a competing risk was estimated at 3.7% (95%CI: 1.4–7.9), 9.6% (95%CI: 5.2–15.6), 20.6% (95%CI: 13.3–29.0), and 28.1% (95%CI: 18.6–38.3, S2 Fig). Most local recurrences occurred relatively late after surgery, while distant metastasis occurred predominantly early after surgery (S2 Fig).

#### Prospective analysis—Overall survival

**Fig 1:**
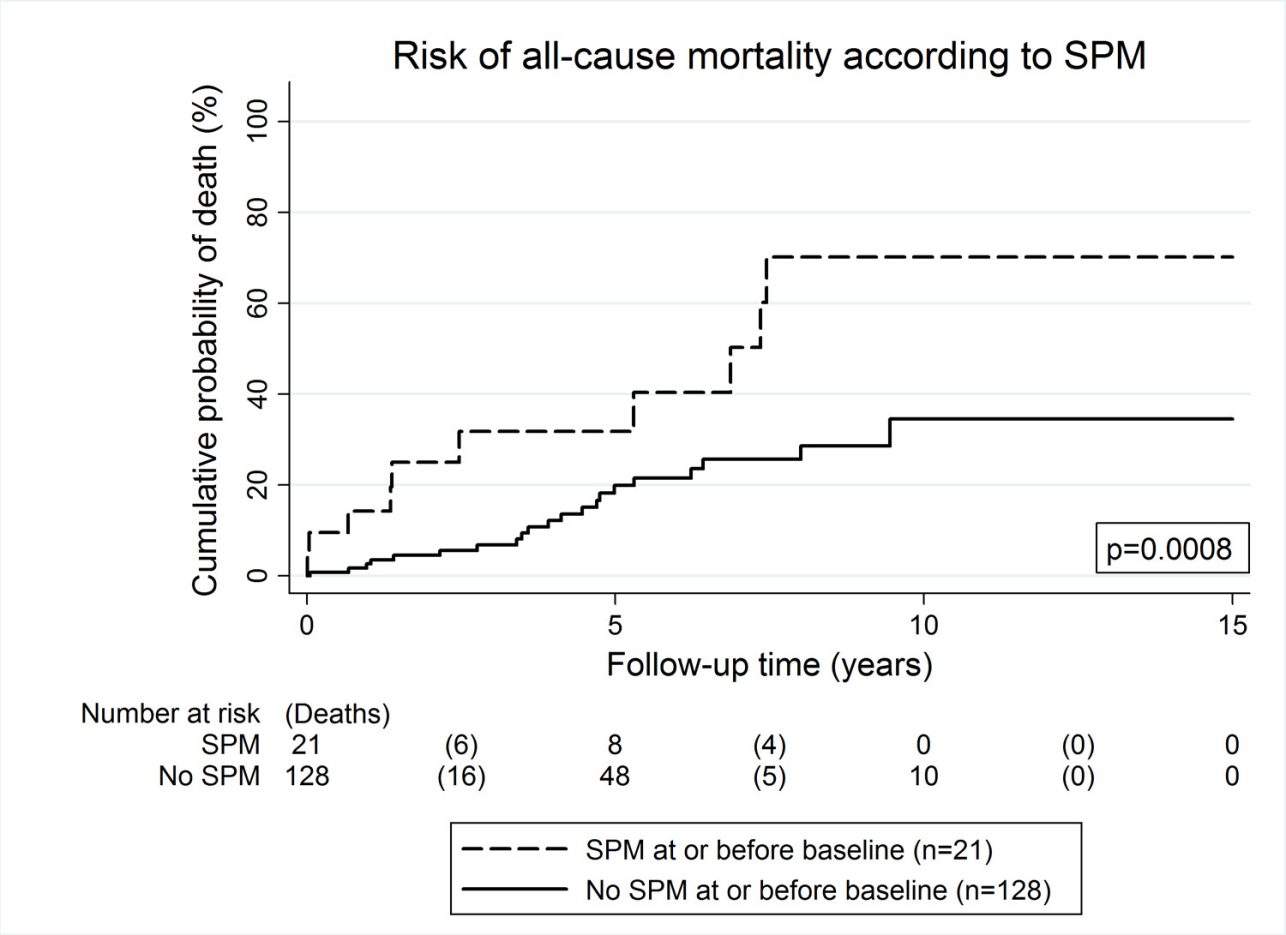
Risk of all-cause mortality according to second primary malignancy status (SPM) at baseline. The presence of a second primary malignancy (SPM) or a history of a SPM at baseline was a significant contributor towards an increased risk of death-from-any-cause. The risk of death was estimated using the inverse Kaplan-Meier estimator, and the boxed p-value using a log-rank test.

**Fig 2:**
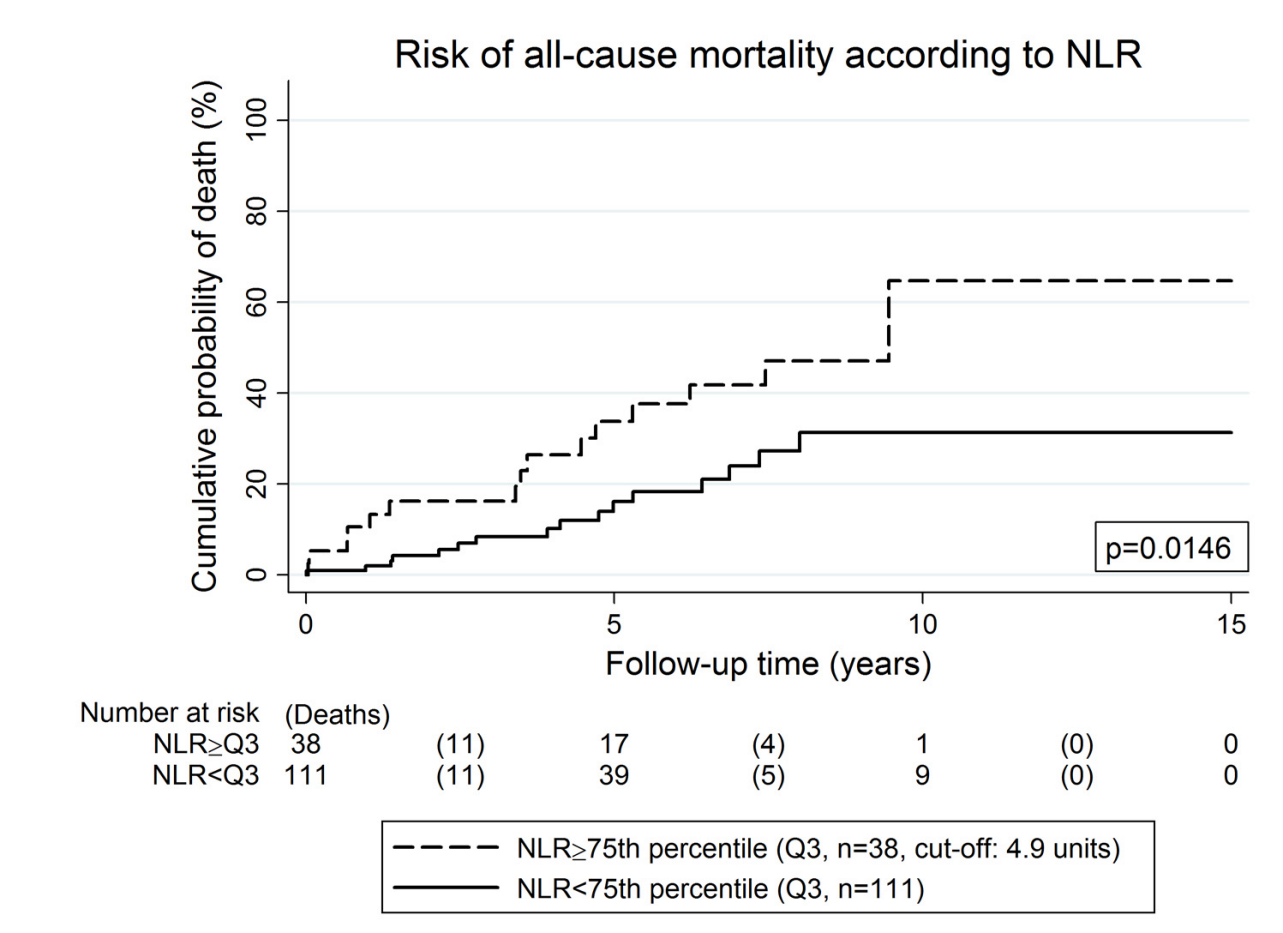
Risk of all-cause mortality according to neutrophil lymphocyte ratio (NLR) at baseline. Patients with an elevated NLR at baseline had a significantly worse overall survival experience. The risk of death was estimated using the inverse Kaplan-Meier estimator, and the boxed p-value using a log-rank test. The NLR was dichotomized into a binary variable at its 75^th^ percentile (i.e. Q3, cut-off: 4.9 units).

Among clinical variables, higher age at entry (Hazard ratio (HR) per 5 years increase = 1.32, 95%CI: 1.10–1.58, p = 0.003) and a SPM at baseline or before (HR = 3.40, 95%CI: 1.59–7.27, p = 0.002, Fig 1) were the strongest predictors of an adverse overall survival experience (Table 2). Further univariable predictors of an unfavorable overall survival were a low Hb level (p = 0.035), a high white blood count (p = 0.007), a high absolute neutrophil count (p = 0.032), a high absolute lymphocyte count (p = 0.008), a high absolute monocyte count (p = 0.003), a high NLR (p = 0.003, Fig 2), and a high PLR (p = 0.004). Adjuvant treatment with imatinib was weakly associated with a lower risk of death (p = 0.12), and the hazard ratio (HR) clearly pointed towards an overall survival benefit with adjuvant treatment (HR = 0.32, 95%CI: 0.08–1.34). Tumor-specific variables such as tumor size, mitotic rate, or a moderate/high risk Miettinen score did not emerge to be univariably associated with overall survival (Table 2).

**Table 2:** Baseline Predictors of Oncologic Outcomes in GIST–Univariable Analysis. The endpoints overall survival and recurrence-free survival were analyzed with Cox proportional hazards models, whereas the endpoints local recurrence, distant metastasis, and recurrence were analyzed using Fine & Gray models. Abbreviations: HR–hazard ratio, SHR–subdistribution hazard ratio, 95%CI– 95% confidence interval, p–p-value, HPF–high power field, g/dL–grams per deciliter, G/L–giga per liter, NLR–neutrophil lymphocyte ratio, dNLR–derived NLR, LMR–lymphocyte monocyte ratio, PLR–platelet lymphocyte ratio.

Variable	Overall survival (OS)			Recurrence-free survival (RFS)			Time-to-recurrence			Time-to-local-recurrence			Time-to-distant-metastasis		
	HR	95%CI	p	HR	95%CI	p	SHR	95%CI	p	SHR	95%CI	p	SHR	95%CI	p
Age at entry(per 5 years increase)	1.32	1.10–1.58	0.003	1.14	1.00–1.31	0.045	0.95	0.84–1.08	0.474	0.85	0.69–1.05	0.135	1.02	0.90–1.17	0.671
Male Gender	0.69	0.34–1.40	0.303	0.91	0.50–1.66	0.766	1.29	0.59–2.84	0.526	0.95	0.22–2.62	0.658	1.07	0.46–2.52	0.875
SPM at baseline or before	3.40	1.59–7.27	0.002	1.48	0.71–3.10	0.290	N/E	N/E	N/E	N/E	N/E	N/E	N/E	N/E	N/E
Tumor size(per 1cm increase)	0.98	0.91–1.06	0.622	1.06	1.01–1.12	0.011	1.10	1.06–1.14	<0.001	1.10	1.02–1.18	0.011	1.09	1.04–1.14	0.001
Mitotic rate >5/50 HPF	1.36	0.67–2.76	0.398	2.95	1.62–5.36	<0.001	9.48	3.67–24.50	<0.001	6.21	1.31–29.46	0.021	8.85	3.04–25.76	<0.001
Adjuvant Treatmentwith Imatinib	0.32	0.08–1.34	0.119	0.60	0.25–1.43	0.250	0.95	0.36–2.49	0.917	1.34	0.28–6.31	0.713	0.64	0.19–2.15	0.473
Haemoglobin(per 1g/dL increase)	0.86	0.74–0.99	0.035	0.87	0.76–0.99	0.032	0.89	0.74–1.07	0.202	0.95	0.65–1.40	0.801	0.88	0.74–1.05	0.146
White Blood Count(per 1G/L increase)	1.10	1.03–1.18	0.007	1.12	1.06–1.20	<0.001	1.04	0.97–1.11	0.256	1.11	1.03–1.20	0.006	0.97	0.87–1.09	0.643
Platelet Count(per 50G/L increase)	1.11	0.92–1.33	0.280	1.20	1.05–1.38	0.009	1.17	1.01–1.36	0.038	1.28	1.03–1.58	0.024	1.08	0.90–1.29	0.398
Absolute Neutrophil Count(per 1G/L increase)	1.09	1.01–1.18	0.032	1.12	1.05–1.20	0.001	1.09	1.01–1.17	0.035	1.19	1.10–1.30	<0.001	1.00	0.90–1.12	0.958
Absolute Lymphocyte Count(per 1G/L increase)	1.30	1.07–1.57	0.008	1.27	1.05–1.53	0.016	0.48	0.20–1.13	0.091	0.89	0.50–1.58	0.688	0.30	0.11–0.80	0.016
Absolute Monocyte Count(per 1G/L increase)	3.34	1.50–7.47	0.003	2.87	1.41–5.82	0.003	1.43	0.52–3.88	0.486	3.47	1.18–10.22	0.024	0.97	0.22–4.23	0.969
NLR(per 1 unit increase)	1.12	1.04–1.20	0.003	1.13	1.06–1.20	<0.001	1.08	1.01–1.16	0.036	1.13	1.03–1.23	0.009	1.05	0.96–1.14	0.294
derived NLR(per 1 unit increase)	1.16	0.97–1.38	0.104	1.27	1.10–1.47	0.001	1.22	1.06–1.42	0.007	1.35	1.12–1.61	0.002	1.11	0.91–1.35	0.289
LMR(per 1 unit increase)	0.87	0.67–1.13	0.297	0.84	0.67–1.05	0.129	0.76	0.57–1.04	0.083	0.67	0.32–1.40	0.291	0.77	0.57–1.02	0.068
PLR(per 50 unit increase)	1.18	1.05–1.33	0.004	1.20	1.09–1.32	<0.001	1.14	1.01–1.28	0.034	1.11	0.97–1.27	0.133	1.13	1.00–1.29	0.053
Miettinen ScoreModerate or High Risk	0.98	0.46–2.07	0.960	2.11	1.12–3.98	0.021	15.67	3.73–65.76	<0.001	7.57	0.92–62.56	0.060	27.54	3.70–205.07	0.001

The association between a higher age and SPM at or before baseline with a higher risk of all-cause-mortality prevailed after including these two variables simultaneously into a multivariable Cox Model. In detail, the model predicted that after adjusting for SPM, one year increase in age at study entry was associated with a 4.7% increase in the risk of death (HR = 1.04, p = 0.015), and after adjusting for age at study entry, SPM at or before baseline was associated with a 2.4-fold increase in the risk of death (HR = 2.36, p = 0.034). In multivariable analysis adjusting for age and SPM, the associations between an increased risk of all-cause-mortality and a low Hb (p = 0.029), a high white blood count (p = 0.004), a high lymphocyte and monocyte count (p = 0.025 and p = 0.020, respectively), and a high NLR/dNLR (p = 0.015) and a high PLR (p = 0.05) prevailed (S2 Table).

#### Prospective analysis–Risk of recurrence

**Fig 3:**
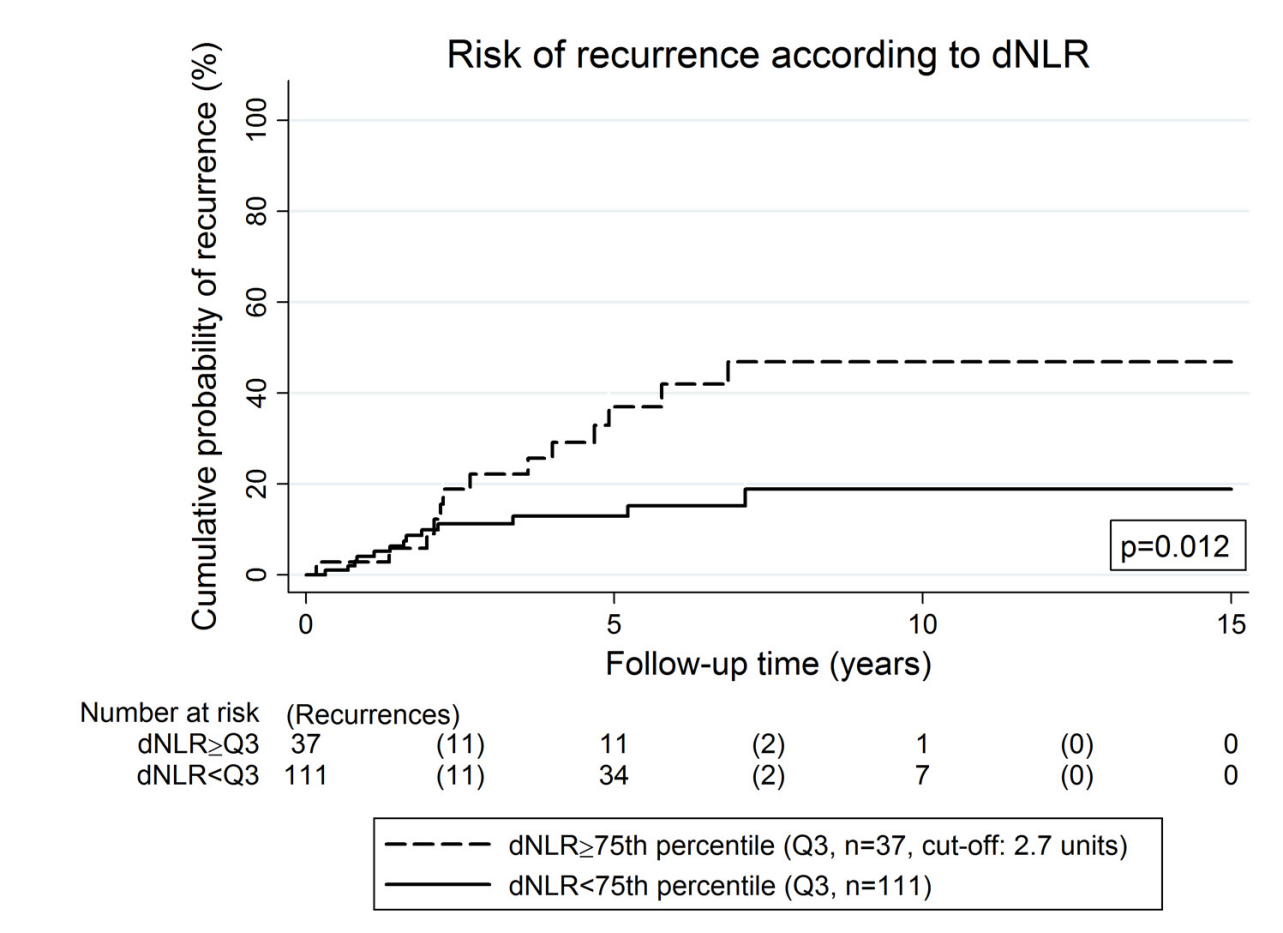
Risk of recurrence according to the derived neutrophil lymphocyte ratio (dNLR) at baseline. Patients with an elevated dNLR at baseline had a significantly higher risk of developing recurrence. The risk of recurrence (defined as a composite of local recurrence and/or distant metastasis, whatever came first) was estimated using competing risk cumulative incidence estimators with death-from-any-cause as the competing event of interest. The boxed p-value was estimated using Gray’s test. The dNLR was dichotomized into a binary variable at its 75^th^ percentile (i.e. Q3, cut-off: 2.7 units).

In univariable competing risk analysis of recurrence risk (a composite of local recurrence and/or distant metastasis accounting for competing mortality), a higher tumor size (p<0.001), a higher mitotic rate (p<0.001) and a moderate/high Miettinen score (p<0.001) were predictors of progression risk. In detail, in patients with a “no risk”, “very low risk”, and “low risk” Miettinen score, the proportion of patients who recurred was below 5%. Conversely, 22% and 42.5% of patients with a “moderate risk” or “high risk” Miettinen score experienced recurrence, respectively. In competing risk regression, the model predicted that in comparison to patients with a “no to low Risk” Miettinen score, patients with a moderate risk score have a 9.7 times higher risk of recurrence (p = 0.008), and patients with a high risk score have an 18.3 times higher risk of recurrence (p<0.001). Among the investigated blood-based biomarkers, a higher NLR (p = 0.036), dNLR (p = 0.007, Fig 3), and PLR were associated with a higher risk of recurrence in univariable competing risk analysis.

In further multivariable analyses, we only considered the Miettinen score, because tumor size and mitotic rate are incorporated in this risk stratification tool. After adjusting for the Miettinen score, the statistically significant univariable associations between the investigated blood-based biomarkers and a higher recurrence risk only prevailed for the NLR/dNLR, but not for the PLR (S3 Table).

#### Prospective analysis–Risk stratification

For recurrence-free survival, a multivariate analysis adjusted for age and Miettinen score was performed. Of all studied variables, a low Hb and an elevated white blood cell count, NLR, dNLR and PLR were associated with a decreased RFS after adjusting for age and Miettinen score (S4 Table). LMR showed no association with RFS in our study population. Next, we added the NLR and PLR to the Miettinen score to evaluate the putative improvement of the prediction of combined disease progression risk and mortality (i.e. RFS) in our patient population. The application of adjuvant therapy was also included into the model for reasons of generalizability. The “base” model including only Miettinen score (HR for moderate/high vs. others = 2.7, 95%CI: 1.4–5.3, p = 0.003) and adjuvant therapy (HR = 0.4, 95%CI: 0.2–1.0, p = 0.05) resulted in a Harrell’s C index of 0.66. After addition of the NLR (adjusted HR per 1 unit increase = 1.1, 95%CI: 1.0–1.2, p = 0.006) or the PLR (adjusted HR per 50 units increase = 1.2, 95%CI: 1.0–1.3, p = 0.006) to this “base” model, Harrell’s C increased to 0.68 and 0.69, respectively. These improvements in discrimination were neither statistically significant for the NLR (difference in Harrell’s C = 0.02, 95%CI: -0.05–0.09, p = 0.527) nor for the PLR (difference in Harrell’s C = 0.03, 95%CI: -0.04–0.11, p = 0.401). Our results demonstrated that the association between NLR and PLR with disease progression was independent of the Miettinen score, but the inclusion of these variables did not reasonably improve the clinical risk prediction of RFS.

#### Sensitivity analysis

Twenty-four (18.6%) patients entered the study before the introduction of adjuvant imatinib to our center. Because trials have shown that imatinib is associated with a strong reduction in the risk of recurrence and death, we examined the impact of the imatinib era on the association between inflammatory biomarkers and recurrence-free survival. We did this by fitting an interaction between the selected biomarkers and a binary variable indicating whether a patient entered our study cohort prior or after the introduction of adjuvant imatinib to our center: Treatment era did not interact with inflammatory markers in any of the conducted analyses (S5 Table), which supports the hypothesis that the inflammatory biomarkers under study remain prognostic for recurrence-free survival also in the imatinib era.

## Discussion

In the present study we examined the clinical course of 149 patients with GIST after curative surgery and found a significant association between low Hb levels, an elevated WBC, NLR, dNLR and decreased OS. For RFS, we identified a low haemoglobin level, an elevated WBC, as well as an elevated NLR, dNLR and PLR as parameters that were significantly associated with poor outcome. Of all studied variables, only elevated dNLR was associated with a higher RR after adjusting for Miettinen score. This initially suggested that this parameter may be used to improve the prediction of disease relapse in this patient population. However, we could then show that the inclusion of NLR or PLR to Miettinen risk score did not reasonably improve the clinical risk prediction of RFS. Notably, SPMs appear to contribute significantly to survival outcome in patients with GIST.

Adjuvant imatinib influences the risk of GIST recurrence over time. The risk of recurrence is high during the first two years after surgery and patients are at a relatively small risk of relapse while therapy is ongoing; however, the risk after finishing therapy with imatinib is substantially increasing. It seems that imatinib delays the risk of recurrence in „high risk”operated GIST patients [29]. In our study, the adjuvant treatment with imatinib was only weakly associated with a lower risk of death but not with the specific clinical endpoints local recurrence and distant metastasis. This result might be due to the fact that patients with a higher risk of local recurrence and distant metastasis were the same patients that received adjuvant imatinib, and adjuvant imatinib thus offset the higher recurrence risk in these patients. In our study population, adjuvant treatment with imatinib was routinely discontinued after 1–3 years of treatment based on the current recommendations. Regarding the generally used Miettinen score we found that in contrast to patients with a “no risk”, “very low risk”, or “low risk” Miettinen score, patients with a “moderate risk” or “high risk” Miettinen score had a dramatically higher risk of local recurrence and distant metastasis. However, we observed no association regarding overall mortality, indicating that the Miettinen score may be very specific for the cancer-specific GIST outcomes rather than OS. The association between NLR and PLR with disease progression was independent of the Miettinen score, but the inclusion of these variables did not improve the clinical risk prediction of 2-yr disease progression and death. This finding indicates that NLR and PLR are independent prognostic variables for RFS, but their impact on improving prognostication in surgical treated GIST patients appears to be limited.

The bi-directional association between inflammation and cancer progression has already been established, and knowledge about the cancer-immune-system-interaction is becoming increasingly important in the era of checkpoint-blockade immunotherapy. Our study group previously investigated the role of blood-based inflammatory biomarkers such as the NLR, PLR, dNLR or LMR in a variety of tumor entities including soft tissue sarcomas (STS), where we demonstrated their role as independent prognostic factors for an adverse survival experience [20–22, 30–32]. However, a recent meta-analysis that focused on the impact of immune cells on clinical outcome suggests that the role of immune cells seems to differ depending on cancer stage and the type of cancer [33]. Lee et al. recently showed that for metastatic GIST patients treated with imatinib, valuable risk factors for OS and progression-free survival (PFS) are the size of the largest metastasis, tumor genotype, primary tumor mitotic count, Hb level and blood neutrophil count [34]. Perez et al. found NLR represents a surrogate for poor prognosis in GIST. They showed a significant correlation between NLR and the mitotic rate (Pearson correlation coefficient, as well as the NLR and tumor size (r = 0.36, p = 0.0001). However, in their population NLR was not independent from the nomogram-predicted outcome (p = 0.12) or Miettinen risk category (p = 0.11) in a multivariate model. Nonetheless they found elevated NLR in tumors larger than 5cm prognostically relevant for RFS (RFS shorter in case of high NLR), which is partly in line with our findings [35]. Racz et al. recently found a high PLR to be associated with reduced RFS but no association between NLR and RFS in GIST patients [36]. This is only partly in line with our results, as we could demonstrate that the elevated PLR associated with a worse RFS. However, we also give evidence to the statistically significant inverse association between elevated NLR and elevated dNLR and OS as well as RFS. A possible explanation for the different results regarding NLR might be, that in the study of Racz patients with adjuvant imatinib had been excluded, and therefore „high risk”patients were not evaluated. The impact of low Hb level on OS and RFS might result from anemia associated co-morbidities that result in decreased survival or from the fact that the grade of anemia is dependent on the extent of cancer-burden [37, 38].

Additionally, we found that the age at study entry and SPM are independent predictors of an adverse survival experience in patients with surgically-resected GIST. When we included both neutrophil and lymphocyte count into a multivariable model with age and SPM, both neutrophil and lymphocyte count remained associated with reduced OS. This suggests that neutrophilia and lymphocytosis may represent separate pathobiologic features of an immune process leading to a higher risk of death. Interestingly, SPM diagnosed at or before baseline emerged as a strong adverse risk factor for OS but not for RFS. This discrepancy between OS and RFS can be explained by the observation that the SPM variable does not appear to be associated with any of the GIST-specific outcomes such as local recurrence and distant metastasis, but rather with a general impairment in overall survival.

There are several limitations to be taken into account. Potential confounding factors such as moderate local or systemic infection, ischemia, acute coronary syndrome, metabolic syndrome, diabetes mellitus and renal or hepatic dysfunction that might affect the white blood cell counts or the haemoglobin level have not been systematically assessed in this study. Finally, the event rate of local recurrence was quite low, which may have led us to “miss” relevant associations between blood-based biomarkers and this endpoint. Moreover, because of the retrospective design of the study we cannot fully exclude a selection bias in our study cohort.

In conclusion, we found that a low Hb level and an elevated WBC, NLR, dNLR and PLR were independently associated with reduced RFS, whereas a low Hb level and an elevated white blood cell count, NLR and dNLR showed a significant association with worse OS in multivariate analysis. However, only dNLR remained statistically significant associated with the tumor-specific endpoint RR. Furthermore, we demonstrated that the addition of the investigated blood-based biomarkers NLR and PLR to Miettienen score revealed no benefit regarding prediction of recurrence and mortality risk. LMR was not associated with clinical outcome in our study population. Synoptically, this paper clarifies the association between blood-based biomarkers and tumor-specific and survival endpoints occurring during long-term follow-up of patients with GIST treated with primary curative surgery.

## Supporting Information

S1 Fig Overall and recurrence-free survival experience of the total cohort. Both functions were estimated using the Kaplan-Meier product limit estimator.
(TIF)

S2 Fig Risk of local recurrence, distant metastasis, and total recurrence in the study population. The risk of all three endpoints was estimated using cumulative incidence functions accounting for all-cause mortality as a competing risk.
(TIF)

S1 Table Rank-based correlations between blood parameters. Reported are Spearman’s correlation coefficients with p-values in round brackets. P-values are from a hypothesis test which tests the null hypothesis that the correlation coefficient is zero. Abbreviations: WBC–white blood cell counts, ANC–neutrophil counts, ALC–lymphocyte counts, AMC–monocyte counts, NLR–neutrophil lymphocyte ratio, dNLR–derived NLR, LMR–lymphocyte monocyte ratio, PLR–platelet lymphocyte ratio.
(DOCX)

S2 Table Baseline predictors of overall survival in GIST–Multivariable analysis adjusted for age and second primary malignancy status at baseline. Results are from Cox models. Abbreviations: HR–hazard ratio, 95%CI– 95% confidence interval, p–p-value, HPF–high power field, g/dL–grams per deciliter, G/L–giga per liter, NLR–neutrophil lymphocyte ratio, dNLR–derived NLR, LMR–lymphocyte monocyte ratio, PLR–platelet lymphocyte ratio.
(DOCX)

S3 Table Baseline predictors of recurrence risk in GIST–Multivariable competing risk analysis adjusted for Miettinen score. Results are from Fine & Gray models. Abbreviations: HR–hazard ratio, 95%CI– 95% confidence interval, p–p-value, HPF–high power field, g/dL–grams per deciliter, G/L–giga per liter, NLR–neutrophil lymphocyte ratio, dNLR–derived NLR, LMR–lymphocyte monocyte ratio, PLR–platelet lymphocyte ratio.
(DOCX)

S4 Table Baseline predictors of recurrence-free survival in GIST–Multivariable analysis adjusted for age and Miettinen score. Results are from Cox models. Abbreviations: HR–hazard ratio, 95%CI– 95% confidence interval, p–p-value, g/dL–grams per deciliter, G/L–giga per liter, NLR–neutrophil lymphocyte ratio, dNLR–derived NLR, LMR–lymphocyte monocyte ratio, PLR–platelet lymphocyte ratio.
(DOCX)

S5 Table Baseline Predictors of Recurrence-free survival (RFS) in GIST–Sensitivity analysis gauging the impact of the imatinib era using interaction analysis. Abbreviations: HR–hazard ratio, 95%CI– 95% confidence interval, p–p-value, g/dL–grams per deciliter, NLR–neutrophil lymphocyte ratio, dNLR–derived NLR, LMR–lymphocyte monocyte ratio, PLR–platelet lymphocyte ratio.
(DOCX)
