# Supplementary figures and images for: Blood-Based Biomarkers Are Associated with Disease Recurrence and Survival in Gastrointestinal Stroma Tumor Patients after Surgical Resection
Source: PLoS One. 2016 Jul 25;11(7):e0159448. doi: 10.1371/journal.pone.0159448 (PMC4959723; doi:10.1371/journal.pone.0159448)

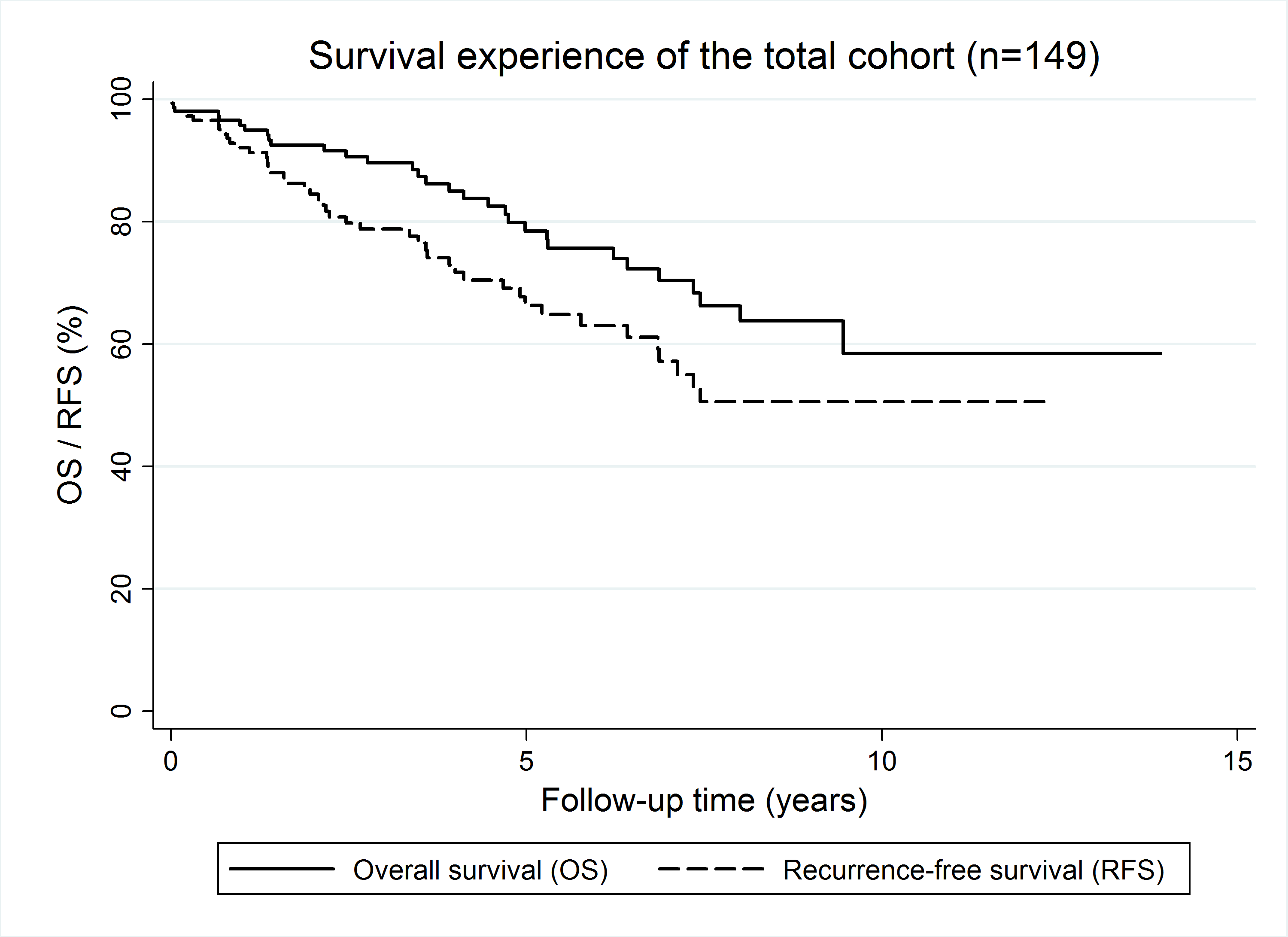

Supplement: S1 Fig — Both functions were estimated using the Kaplan-Meier product limit estimator. (TIF) [file pone.0159448.s001.tif]

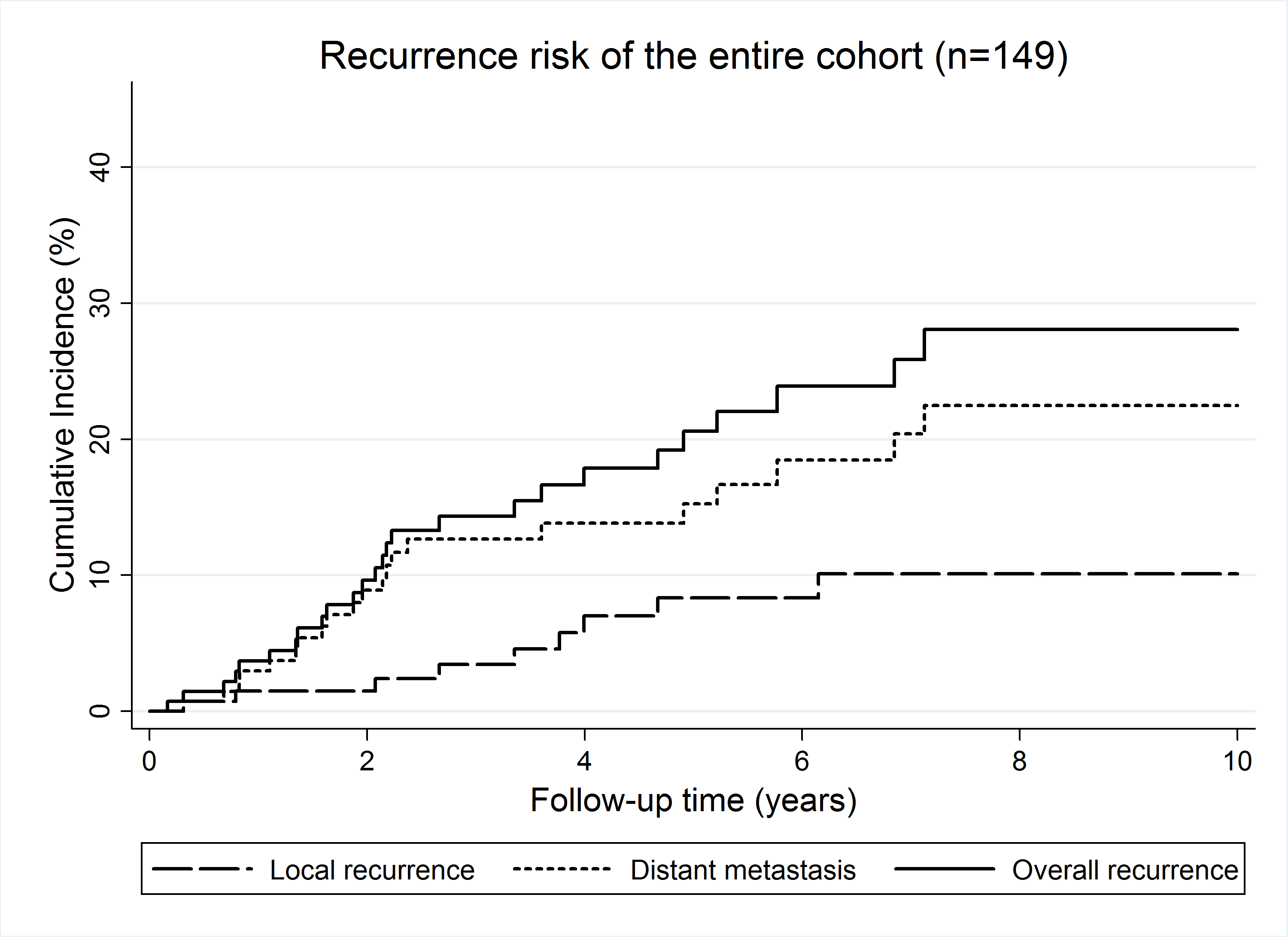

Supplement: S2 Fig — The risk of all three endpoints was estimated using cumulative incidence functions accounting for all-cause mortality as a competing risk. (TIF) [file pone.0159448.s002.tif]
