# Supplementary material for: Blood-Based Biomarkers Are Associated with Disease Recurrence and Survival in Gastrointestinal Stroma Tumor Patients after Surgical Resection
Source: PLoS One. 2016 Jul 25;11(7):e0159448. doi: 10.1371/journal.pone.0159448 (PMC4959723; doi:10.1371/journal.pone.0159448)
Supplement: S1 Table — Reported are Spearman’s correlation coefficients with p-values in round brackets. P-values are from a hypothesis test which tests the null hypothesis that the correlation coefficient is zero. Abbreviations: WBC–white blood cell counts, ANC–neutrophil counts, ALC–lymphocyte counts, AMC–monocyte counts, NLR–neutrophil lymphocyte ratio, dNLR–derived NLR, LMR–lymphocyte monocyte ratio, PLR–platelet lymphocyte ratio. (DOCX) [file pone.0159448.s003.docx]

|  | WBC | ANC | ALC | AMC | NLR | dNLR | PLR | LMR |
| --- | --- | --- | --- | --- | --- | --- | --- | --- |
| WBC | 1.00 |  |  |  |  |  |  |  |
| ANC | 0.94  (<0.0001) | 1.00 |  |  |  |  |  |  |
| ALC | 0.21  (0.009) | 0.00  (0.972) | 1.00 |  |  |  |  |  |
| AMC | 0.70  (<0.0001) | 0.64  (<0.0001) | 0.14  (0.084) | 1.00 |  |  |  |  |
| NLR | 0.57  (<0.0001) | 0.76  (<0.0001) | -0.59  (<0.0001) | 0.40  (<0.0001) | 1.00 |  |  |  |
| dNLR | 0.57 (<0.0001) | 0.78  (<0.0001) | -0.50  (<0.0001) | 0.31  (0.0001) | 0.96  (<0.0001) | 1.00 |  |  |
| PLR | 0.05  (0.532) | 0.18  (0.026) | -0.70  (<0.0001) | 0.10  (0.229) | 0.60  (<0.0001) | 0.50  (<0.0001) | 1.00 |  |
| LMR | -0.36  (<0.0001) | -0.47  (<0.0001) | 0.60  (<0.0001) | -0.64  (<0.0001) | -0.76  (<0.0001) | -0.63  (<0.0001) | -0.63  (<0.0001) | 1.00 |
