# Supplementary material for: Blood-Based Biomarkers Are Associated with Disease Recurrence and Survival in Gastrointestinal Stroma Tumor Patients after Surgical Resection
Source: PLoS One. 2016 Jul 25;11(7):e0159448. doi: 10.1371/journal.pone.0159448 (PMC4959723; doi:10.1371/journal.pone.0159448)
Supplement: S2 Table — Results are from Cox models. Abbreviations: HR–hazard ratio, 95%CI– 95% confidence interval, p–p-value, HPF–high power field, g/dL–grams per deciliter, G/L–giga per liter, NLR–neutrophil lymphocyte ratio, dNLR–derived NLR, LMR–lymphocyte monocyte ratio, PLR–platelet lymphocyte ratio. (DOCX) [file pone.0159448.s004.docx]

| **Variable** |  | **Overall Survival** | | |
| --- | --- | --- | --- | --- |
|  |  | **HR** | **95%CI** | **p** |
|  |  |  |  |  |
| Male Gender |  | 0.70 | 0.34-1.43 | 0.325 |
| Tumor size  (per 1cm increase) |  | 1.00 | 0.93-1.08 | 0.945 |
| Mitotic rate >5/50 HPF |  | 2.17 | 0.95-4.97 | 0.066 |
| Adjuvant Treatment  with Imatinib |  | 0.47 | 0.11-2.03 | 0.313 |
| Haemoglobin  (per 1g/dL increase) |  | 0.83 | 0.71-0.98 | 0.029 |
| White Blood Count  (per 1G/L increase) |  | 1.10 | 1.03-1.19 | 0.004 |
| Platelet Count  (per 50G/L increase) |  | 1.08 | 0.91-1.29 | 0.370 |
| Absolute Neutrophil Count  (per 1G/L increase) |  | 1.09 | 1.01-1.19 | 0.034 |
| Absolute Lymphocyte Count  (per 1G/L increase) |  | 1.27 | 1.03-1.57 | 0.025 |
| Absolute Monocyte Count  (per 1G/L increase) |  | 2.60 | 1.16-5.81 | 0.020 |
| NLR  (per 1 unit increase) |  | 1.10 | 1.02-1.19 | 0.015 |
| derived NLR  (per 1 unit increase) |  | 1.22 | 1.01-1.47 | 0.037 |
| LMR  (per 1 unit increase) |  | 0.97 | 0.74-1.27 | 0.827 |
| PLR  (per 50 unit increase) |  | 1.12 | 1.00-1.26 | 0.051 |
| Miettinen Score  Moderate or High Risk |  | 1.37 | 0.60-3.19 | 0.456 |
