# Supplementary material for: Blood-Based Biomarkers Are Associated with Disease Recurrence and Survival in Gastrointestinal Stroma Tumor Patients after Surgical Resection
Source: PLoS One. 2016 Jul 25;11(7):e0159448. doi: 10.1371/journal.pone.0159448 (PMC4959723; doi:10.1371/journal.pone.0159448)
Supplement: S3 Table — Results are from Fine & Gray models. Abbreviations: HR–hazard ratio, 95%CI– 95% confidence interval, p–p-value, HPF–high power field, g/dL–grams per deciliter, G/L–giga per liter, NLR–neutrophil lymphocyte ratio, dNLR–derived NLR, LMR–lymphocyte monocyte ratio, PLR–platelet lymphocyte ratio. (DOCX) [file pone.0159448.s005.docx]

| **Variable** |  | **Time-to-Recurrence** | | |
| --- | --- | --- | --- | --- |
|  |  | **HR** | **95%CI** | **p** |
|  |  |  |  |  |
| Male Gender |  | 0.78 | 0.35-1.76 | 0.555 |
| Adjuvant Treatment  with Imatinib |  | 0.40 | 0.15-1.04 | 0.061 |
| Haemoglobin  (per 1g/dL increase) |  | 0.93 | 0.74-1.17 | 0.553 |
| White Blood Count  (per 1G/L increase) |  | 1.04 | 0.93-1.16 | 0.485 |
| Platelet Count  (per 50G/L increase) |  | 1.08 | 0.88-1.31 | 0.468 |
| Absolute Neutrophil Count  (per 1G/L increase) |  | 1.08 | 0.96-1.21 | 0.204 |
| Absolute Lymphocyte Count  (per 1G/L increase) |  | 0.61 | 0.21-1.82 | 0.380 |
| Absolute Monocyte Count  (per 1G/L increase) |  | 1.04 | 0.36-3.02 | 0.947 |
| NLR  (per 1 unit increase) |  | 1.10 | 0.99-1.23 | 0.080 |
| derived NLR  (per 1 unit increase) |  | 1.23 | 1.00-1.52 | 0.048 |
| LMR  (per 1 unit increase) |  | 0.90 | 0.64-1.26 | 0.522 |
| PLR  (per 50 unit increase) |  | 1.11 | 0.95-1.31 | 0.197 |
| SPM at or before baseline |  | N/E | N/E | N/E |
| Age at study entry  (per 5 years increase) |  | 1.01 | 0.82-1.24 | 0.927 |
