# Supplementary material for: Blood-Based Biomarkers Are Associated with Disease Recurrence and Survival in Gastrointestinal Stroma Tumor Patients after Surgical Resection
Source: PLoS One. 2016 Jul 25;11(7):e0159448. doi: 10.1371/journal.pone.0159448 (PMC4959723; doi:10.1371/journal.pone.0159448)
Supplement: S4 Table — Results are from Cox models. Abbreviations: HR–hazard ratio, 95%CI– 95% confidence interval, p–p-value, g/dL–grams per deciliter, G/L–giga per liter, NLR–neutrophil lymphocyte ratio, dNLR–derived NLR, LMR–lymphocyte monocyte ratio, PLR–platelet lymphocyte ratio. (DOCX) [file pone.0159448.s006.docx]

| **Variable** |  | **Recurrence-free survival** | | |
| --- | --- | --- | --- | --- |
|  |  | **HR** | **95%CI** | **p** |
|  |  |  |  |  |
| Male Gender |  | 0.74 | 0.40-1.38 | 0.345 |
| Adjuvant Treatment  with Imatinib |  | 0.43 | 0.17-1.08 | 0.073 |
| Haemoglobin  (per 1g/dL increase) |  | 0.86 | 0.74-1.00 | 0.049 |
| White Blood Count  (per 1G/L increase) |  | 1.14 | 1.06-1.23 | 0.001 |
| Platelet Count  (per 50G/L increase) |  | 1.17 | 1.00-1.37 | 0.044 |
| Absolute Neutrophil Count  (per 1G/L increase) |  | 1.14 | 1.04-1.25 | 0.004 |
| Absolute Lymphocyte Count  (per 1G/L increase) |  | 1.34 | 1.09-1.64 | 0.005 |
| Absolute Monocyte Count  (per 1G/L increase) |  | 2.41 | 1.20-4.84 | 0.013 |
| NLR  (per 1 unit increase) |  | 1.12 | 1.03-1.22 | 0.007 |
| derived NLR  (per 1 unit increase) |  | 1.22 | 1.01-1.49 | 0.043 |
| LMR  (per 1 unit increase) |  | 0.95 | 0.75-1.20 | 0.647 |
| PLR  (per 50 unit increase) |  | 1.14 | 1.02-1.28 | 0.024 |
| SPM at or before baseline |  | 1.94 | 0.88-4.31 | 0.102 |
