# Supplementary material for: Blood-Based Biomarkers Are Associated with Disease Recurrence and Survival in Gastrointestinal Stroma Tumor Patients after Surgical Resection
Source: PLoS One. 2016 Jul 25;11(7):e0159448. doi: 10.1371/journal.pone.0159448 (PMC4959723; doi:10.1371/journal.pone.0159448)
Supplement: S5 Table — Abbreviations: HR–hazard ratio, 95%CI– 95% confidence interval, p–p-value, g/dL–grams per deciliter, NLR–neutrophil lymphocyte ratio, dNLR–derived NLR, LMR–lymphocyte monocyte ratio, PLR–platelet lymphocyte ratio. (DOCX) [file pone.0159448.s007.docx]

| **Variable** |  | **Univariable analysis** | | | **Multivariable analysis** | | |
| --- | --- | --- | --- | --- | --- | --- | --- |
|  |  | **HR** | **95%CI** | **p** | **HR** | **95%CI** | **p** |
|  |  |  |  |  |  |  |  |
| Haemoglobin  (per 1g/dL increase) |  | 0.86 | 0.77-0.99 | 0.031 | 0.91 | 0.79-1.06 | 0.228 |
| Study entry prior Jan, 1^st^, 2014 |  | N/A | N/A | N/A | 28.2 | 0.58-1370.1 | 0.092 |
| Interaction |  | N/A | N/A | N/A | 0.77 | 0.55-1.07 | 0.123 |
| White Blood Count  (per 1G/L increase) |  | 1.13 | 1.06-1.20 | <0.0001 | 1.14 | 1.07-1.21 | <0.0001 |
| Study entry prior Jan, 1^st^, 2014 |  | N/A | N/A | N/A | 3.07 | 0.43-21.9 | 0.264 |
| Interaction |  | N/A | N/A | N/A | 0.90 | 0.69-1.16 | 0.415 |
| NLR  (per 1 unit increase) |  | 1.13 | 1.06-1.20 | <0.0001 | 1.15 | 1.08-1.23 | <0.0001 |
| Study entry prior Jan, 1^st^, 2014 |  | N/A | N/A | N/A | 2.11 | 0.72-6.19 | 0.173 |
| Interaction |  | N/A | N/A | N/A | 0.89 | 0.75-1.07 | 0.213 |
| derived NLR  (per 1 unit increase) |  | 1.27 | 1.10-1.47 | 0.001 | 1.32 | 1.13-1.53 | <0.0001 |
| Study entry prior Jan, 1^st^, 2014 |  | N/A | N/A | N/A | 2.48 | 0.63-9.83 | 0.195 |
| Interaction |  | N/A | N/A | N/A | 0.78 | 0.48-1.28 | 0.329 |
| LMR  (per 1 unit increase) |  | 0.84 | 0.67-1.05 | 0.129 | 0.86 | 0.67-1.11 | 0.252 |
| Study entry prior Jan, 1^st^, 2014 |  | N/A | N/A | N/A | 1.43 | 0.33-6.27 | 0.635 |
| Interaction |  | N/A | N/A | N/A | 0.90 | 0.52-1.58 | 0.720 |
| PLR  (per 50 unit increase) |  | 1.19 | 1.09-1.31 | <0.0001 | 1.20 | 1.08-1.32 | <0.0001 |
| Study entry prior Jan, 1^st^, 2014 |  | N/A | N/A | N/A | 1.25 | 0.27-5.75 | 0.772 |
| Interaction |  | N/A | N/A | N/A | 1.00 | 0.76-1.32 | 0.989 |
